# Growth, filtration and respiration characteristics of small single-osculum demosponge *Halichondria panicea* explants

**DOI:** 10.1242/jeb.247132

**Published:** 2024-04-19

**Authors:** Hans Ulrik Riisgård, Florian Lüskow, Poul S. Larsen

**Affiliations:** ^1^ Marine Biological Research Centre, Department of Biology, University of Southern Denmark, 5300 Kerteminde, Denmark; ^2^ Department of Earth, Ocean and Atmospheric Sciences, University of British Columbia, 2039-2207 Main Mall, Vancouver, BC, Canada, V6T 1Z4; ^3^ DTU Construct, Technical University of Denmark, 2800 Kgs Lyngby, Denmark

**Keywords:** Allometric scaling, Sponge module, Filtration rate, Respiration rate, Growth

## Abstract

Filter-feeding demosponges are modular organisms that consist of modules each with one water-exit osculum. Once a mature module has been formed, the weight-specific filtration and respiration rates do not change. Sponge modules only grow to a certain size and for a sponge to increase in size, new modules must be formed. However, the growth characteristics of a small single-osculum module sponge are fundamentally different from those of multi-modular sponges, and a theoretically derived volume-specific filtration rate scales as *F*/*V*=*V*^−1/3^, indicating a decrease with increasing total module volume (*V*, cm^3^). Here, we studied filtration rate (*F*, l h^−1^), respiration rate (*R*, ml O_2_ h^−1^), volume-specific (*F*/*V*) and weight-specific (*F*/*W*) filtration rates, and the ratios *F*/*R* and *F*/*W* along with growth rates of small single-osculum demosponge *Halichondria panicea* explants of various sizes exposed to various concentrations of algal cells. The following relationships were found: *F*/*V*=7.08*V*^−0.24^, *F*=*a*_1_*W*^1.05^, and *R*=*a*_2_*W*^0.68^ where *W* is the dry weight (mg). The *F*/*R* and *F*/*W* ratios were constant and essentially independent of *W*, and other data indicate exponential growth. It is concluded that the experimental data support the theoretical *F*/*V*∝*V*^–1/3^.

## Teaser

**Summary:** Sponges are modular organisms and in order to grow, new modules must be formed, but the growth of a single module is fundamentally different, with filtration rate scaling to module volume as *F*/*V*∝*V*^1/3^.

## INTRODUCTION

All demosponges, except a few carnivorous ones (Vacelet and Boury-Esnault, 1995; Lee et al., 2012), are filter-feeding modular organisms that consist of a set of repetitive aquiferous units or modules with one water-exit osculum per module (Fry, 1970; Ereskovskii, 2002; Kealy et al., 2019; Kumala et al., 2023). However, some large tropical single-osculum sponges are composed of many modules, each with a true osculum that opens into a common spongocoel, which opens to the ambient water via a static pseudo-osculum (Riisgård and Larsen, 2022b). Once a mature module has been formed, its filtration (*F*) and respiration (*R*) rates no longer change over time. Therefore, when a sponge grows to become a large ‘population of modules’, both the total filtration and respiration rates of the sponge increase linearly with the increasing number of mature modules, i.e. *F=a*_1_*W^b^*^_1_^ and *R*=*a*_2_*W^b^*^_2_^ where *b*_1_=*b*_2_=1. According to the bioenergetics growth model presented by Riisgård and Larsen (2022a), the weight-specific growth rate µ=(1/*W*)d*W*/d*t*=*aW^b^*^_1_^/*W*, which for *b*_1_=1 becomes µ=*a*, which is a constant and the growth is therefore exponential according to definition.

Sponge modules only grow to a certain size because of increasing resistance to flow in the increasingly longer inhalant and exhalant canals. Further, an increasing relative content of water compared with sponge-body tissue puts an upper limit on the size of the module for it to remain a stable structure. Thus, for a sponge to increase in size, new modules must be formed, which leads to a multi-oscular sponge that consists of single-oscular modules that have reached their maximal size and hence their maximal filtration rates. This implies the scaling *F*/*V* is constant, because the total sponge volume equals *V*=number of modules×volume of module (see fig. 3 of Kumala et al., 2023). This also implies that both filtration and respiration rates increase linearly with *V* in multi-oscular sponges. However, the growth characteristics of a small single-osculum module sponge are fundamentally different.

Reiswig (1975) studied the aquiferous system elements in the marine demosponges *Halichondria panicea* and *Haliclona permollis* and noticed variations in tissue densities and canal systems near ‘growth points’ (tips of tubular units or branches) compared with ‘mature’ regions with relatively stable dimensions. We believe that small growing single-osculum single-module explants of *H. panicea* are comparable to ‘growth points’ in the large multi-oscula multi-modular sponge. In this way by using single-osculum explants, we can study the growth, filtration and respiration characteristics of sponge ‘growth points’, which is of fundamental importance for understanding the growth and bioenergetics of sponges.

A scaling relationship between filtration rate (*F*) and body volume (*V*) of a single-osculum module was derived by Riisgård and Larsen (2022b) by considering a single inhalant canal of length *L* whose wall with water-pumping choanocyte chambers (CCs) separates it from the exhalant canal system. The filtration rate created by the CCs is determined by the product of the filtration rate (*F*_CC_) of each CC and their number, which is proportional to the wall area, i.e. *F*∝*L*^2^, while the volume scales as *V*∝*L*^3^ from which it follows that *F*∝(*V*^1/3^)^2^ or *F=aV*^2/3^. Hence, the volume-specific filtration rate scales as *F*/*V*∝*V*^2/3–1^=*V*^–1/3^ (Riisgård and Larsen, 2022b), indicating a decrease with increasing *V* when a small single-osculum sponge module grows bigger. The predicted scaling was supported by data from the literature of experimentally measured *F* and *V* in single-osculum *H. panicea* explants (see fig. 2 of Riisgård and Larsen, 2022b). Here, we further studied filtration and respiration rates, volume-specific and weight-specific filtration rates, *F*/*R* ratios and body growth rates of small single-osculum *H. panicea* demosponge explants of various sizes exposed to different concentrations of algal cells.

It has recently been pointed out by Riisgård and Larsen (2022a) that in filter-feeding marine invertebrates, the *F*/*R* ratio is generally high (i.e. >10 l of water filtered per ml oxygen consumed) in filter-feeding marine invertebrates and that the oxygen extraction efficiency is consequently low, typically ≤1% (Jørgensen et al., 1986). Riisgård and Larsen (2022a) suggested that when the biomass of a growing filter feeder increases, the total respiration consequently increases, but the increasing oxygen demand is easily met by an increase in oxygen extraction efficiency. However, a growing animal must also increase its filtration rate, and thus the ingestion of food to cover its growth and respiratory needs. This ensures that the *F*/*R* ratio remains unchanged because a reduction in the *F*/*R* ratio would cause starvation. Therefore, Riisgård and Larsen (2022a) suggested – as a theory – that the *b*-exponents in the equations for filtration and respiration, *F=a*_1_*W^b^*^_1_^* *and *R=a*_2_*W^b^*^_2_^, may (during evolution) have become near equal depending on species and adaptation to living site. Because of the simple structure of sponges, which have no organs or real tissue that may store energy reserves to overcome starvation periods, the exponents for *F* and *R* versus *W* tend to be close to 1 as seen in multi-oscular multi-module sponges (Reiswig, 1974; Thomassen and Riisgård, 1995; Southwell et al., 2008; Fiore et al., 2013; McMurray et al., 2014; Ludeman et al., 2017; Goldstein et al., 2019; Dahihande and Thakur, 2019; Kumala et al., 2023; Lesser, 2023). However, little is known about *b*-exponents in small growing single-osculum sponges, and therefore one objective of the present study was to test the above hypothesis of *b*_1_≈*b*_2_≈1 by means of experimental data.

It is well known that sponges may undergo periodic cessation of filtering activity (Reiswig, 1971) or reduce filtration activity as a result of disturbance (Riisgård et al., 2016), but the *b*-exponents presented in the present study are based on optimally filtering sponges.

## MATERIALS AND METHODS

### Collection of sponges and growth experiments

**Fig. 1.**
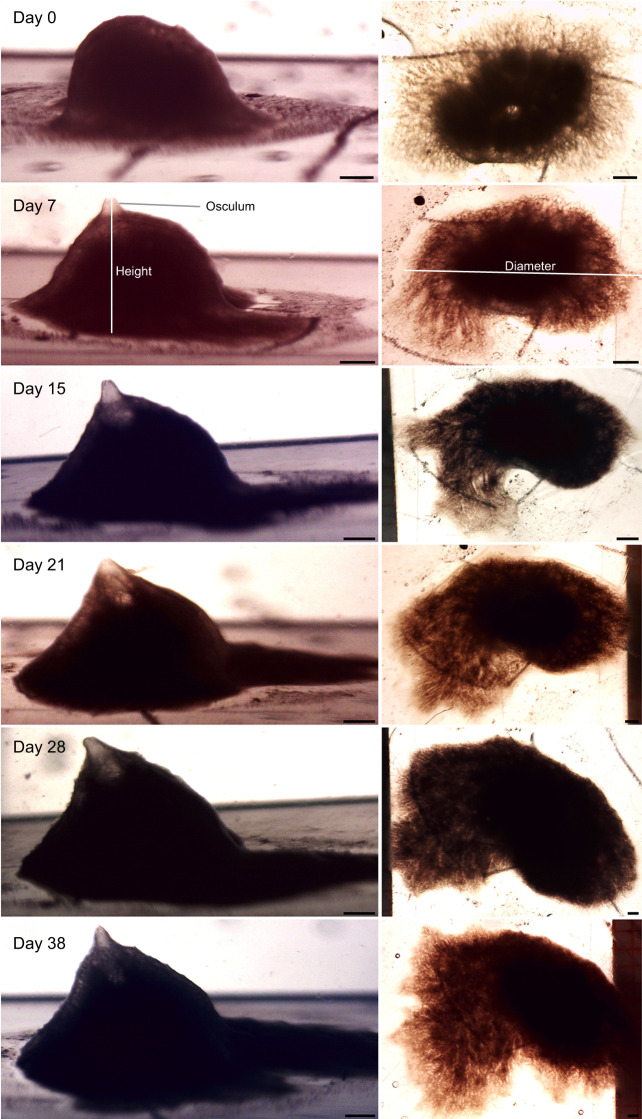
**Growth of a *Halichondria panicea* sponge module fed with *Rhodomonas salina.*** Mean *R. salina* concentration was 7510±2438 cells ml^−1^ [corresponding to 9.4±3.1 µg chlorophyll *a* (Chl *a*) l^−1^, treatment 10 in Table 1]. The diameter was measured from the top view (right column) and height from the side view (left column) picture. Scale bars: 1 mm.

Specimens of the demosponge *Halichondria panicea* (Pallas 1766) were collected in the inlet to Kerteminde Fjord, Denmark (55°26′59″N, 10°39′41″E) in January 2018 and transported to the nearby Marine Biological Research Centre in Kerteminde (University of Southern Denmark). Here, 7 donor sponges (time interval *t*=17 days, temperature *T*=6.5±1.2°C, salinity *S*=14.9±2.0, means±s.d.) were cleaned of epifauna and processed to single-osculum explants (hereafter ‘modules’) as described by Kumala et al. (2017). Modules were placed in object slide racks in 5 l aquaria with 38 µm filtered seawater. Temperature and salinity (*T*=11.7±2.4°C, *S*=18.7±1.2; Fig. S1) were measured daily using a YSI 30 salinity, conductivity and temperature system (Yellowstone Scientific Instruments, Big Sky, MT, USA). To study module growth as a function of food concentration, the size of the modules (e.g. no. 46 at *t*=0–38 days, Fig. 1) was monitored over time under six food treatments: 0, 1, 2, 3, 5 and 10 µg chlorophyll *a* (Chl *a*) l^−1^ (*n*=11–18 per treatment) chosen within the natural range of Danish coastal waters with a median value of 5.1 Chl *a* l^−1^ (Clausen and Riisgård, 1996). The Chl *a* concentration in the aquaria was adjusted by the addition of algal suspension (*Rhodomonas salina*) from a batch culture. To maintain the algal concentrations within a certain known range, new algal suspension was added daily to the experimental tanks, whereas the water was renewed twice a week. Every 24 h, the concentration of *R. salina* (*C*, cells ml^−1^) was measured with an Elzone 5380 Particle Size Analyzer (Micromeritics Instrument Corporation, Norcross, GA, USA). Based on the volume of the experimental chamber (*V*_ex_, ml) and the initial (*C*_0_) and final (*C_t_*) algal concentration, total filtration rates of all sponge modules per treatment (*F*, l h^−1^) were determined as (Coughlan, 1969; Riisgård, 2001):
(1)

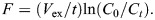
The corresponding mean concentration *C*_m_ was calculated as (Riisgård et al., 2016):
(2)

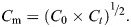


**Table 1.**
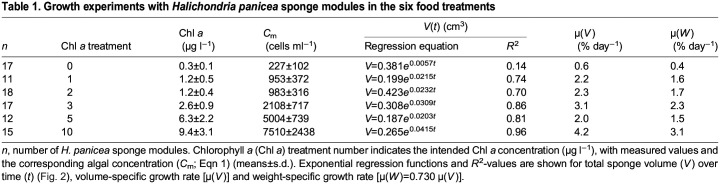
Growth experiments with *Halichondria panicea* sponge modules in the six food treatments

### Growth rate

For size measurements, top and side view images of modules were taken once a week using a stereomicroscope (Leica MZ12.5) with an integrated digital camera (Leica IC80 HD). Images were analysed using ImageJ (version 1.48v). The total sponge volume per treatment (sum of all sponges in a batch of *n* modules; *V*, cm^3^) was determined from module volume (*V*_mod_, mm^3^ module^−1^) based on the modified cone equation (Goldstein et al., 2019):
(3)

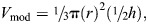
where *r* (mm) describes the top-view radius and *h* (mm) the side-view height of flat near-circular sponge explants. The validity of the assumption of a cone-shaped sponge module was tested by comparing estimated volumes (Eqn 3) with measured volumes using a precision balance (Sartorius BP210D). Further, wet weight (*W*_wet_, mg) versus dry weight (*W*, mg) of sponge modules was determined.

Discrete values of weight-specific growth rate were estimated for growth from *W*_0_ to *W_t_* during the period Δ*t*=*t*−0 as (Riisgård et al., 2014):
(4)

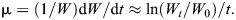


The mean value of µ over a time period was determined as the coefficient in the exponent of an exponential regression to a plot of total sponge module volume (*V*) versus *t*. For a given growth period, the corresponding average dry weight was determined as:
(5)

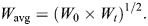


### Respiration rate

The oxygen consumption of sponge modules under different food treatments (0, 1, 2, 3, 5 and 10 µg Chl *a* l^−1^) was measured using an optical oxygen meter system (FireStingO_2_, Pyro Science, Aachen, Germany). Prior to measurements, optical fibres were attached to contactless oxygen sensor spots in the respiration chambers and two-point calibrated to 100% air-saturated (using air bubbling) and fully deoxygenated (by addition of Na_2_S_2_O_4_) filtered seawater. All modules from each treatment group (*n*=11–18 modules) were placed in a closed respiration chamber (*V*_ex_=165±11 ml) with sterile filtered (0.2 µm) seawater (salinity and temperature as in the cultivation tanks). Respiration chambers with and without (control) sponge modules were submerged in a water bath at room temperature under constant stirring. The dissolved oxygen concentration (mg O_2_ l^−1^) was recorded in 1 s intervals for 60 min using Pyro Oxygen Logger^©^ software. Temperature changes were compensated for through an external temperature sensor submerged in the water bath. The total respiration rate (*R*, µg O_2_ h^−1^) of all modules per food treatment was determined from the removal of dissolved oxygen (*b*, µg O_2_ l^−1^ min^−1^) in the respiration chamber of a given volume (*V*_ex_, l) over time as:
(6)

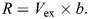


### Conversion factors

Because *in situ* (wet) sponge modules of volume *V* have a density essentially equal to that of seawater, the volume *V*∝*W*_wet_ (wet weight) and their dry weight (*W*) were calculated by the power law relationship *W*(*W*_wet_). Conversion of algal cells to chlorophyll *a* concentration was calculated as 1 µg Chl *a* l^−1^=799 *R. salina* cells ml^−1^ (Clausen and Riisgård, 1996).

## RESULTS

**Fig. 2.**
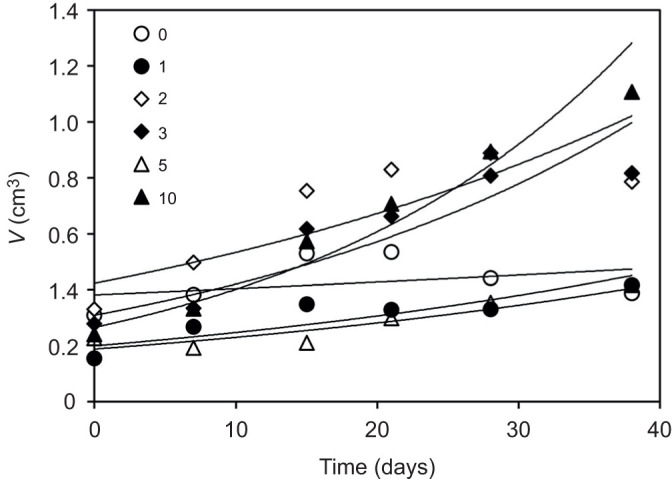
**Growth of *H. panicea* sponge modules over time for the different food treatments.** Volume (*V*) versus time and exponential regression functions (see Table 1 for equations) are shown for the six food treatments: 0, 1, 2, 3, 5 and 10 µg Chl *a* l^−1^. Data from Table S1.

**Fig. 3.**
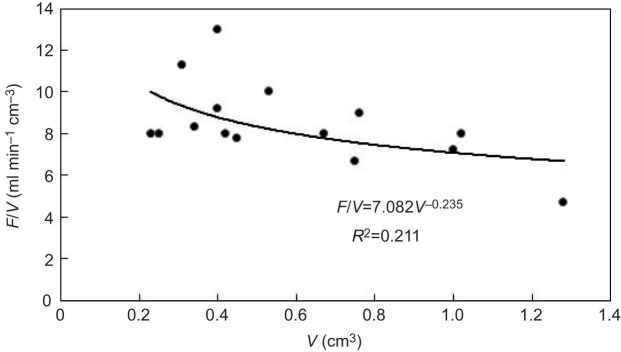
**Relationship between volume-specific filtration rate (*F*) and volume (*V*) of *H. panicea* sponge modules.** Data from Table 2.

**Fig. 4.**
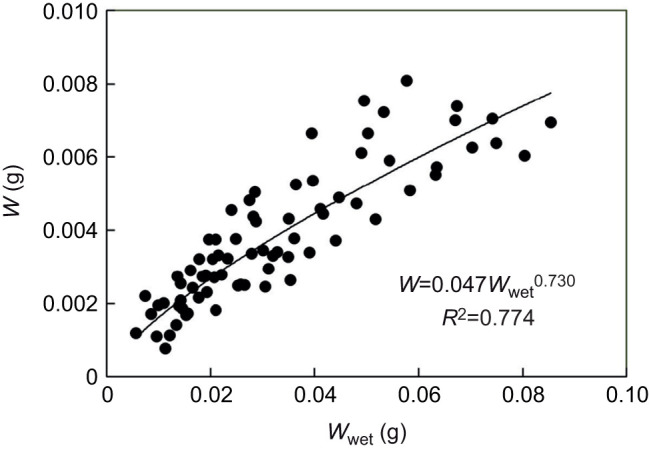
Relationship between dry weight (*W*) and wet weight (*W*_wet_) of *H. panicea* sponge modules.

**Fig. 5.**
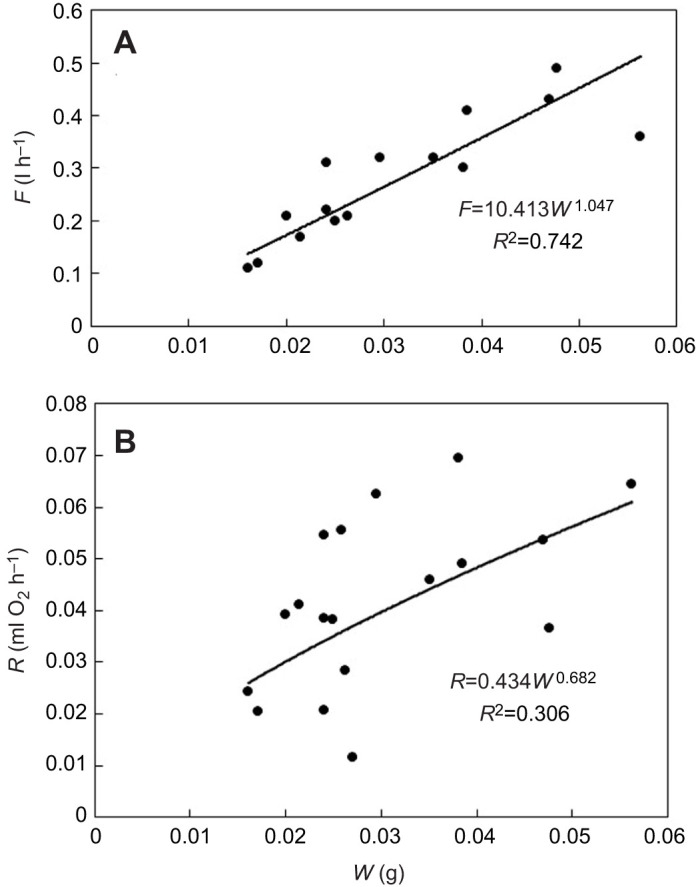
**Filtration rate and respiration rate versus and dry weight of *H. panicea* sponge modules.** (A) Filtration rate (*F*). (B) Respiration rate (*R*). Data from Table 2.

**Fig. 6.**
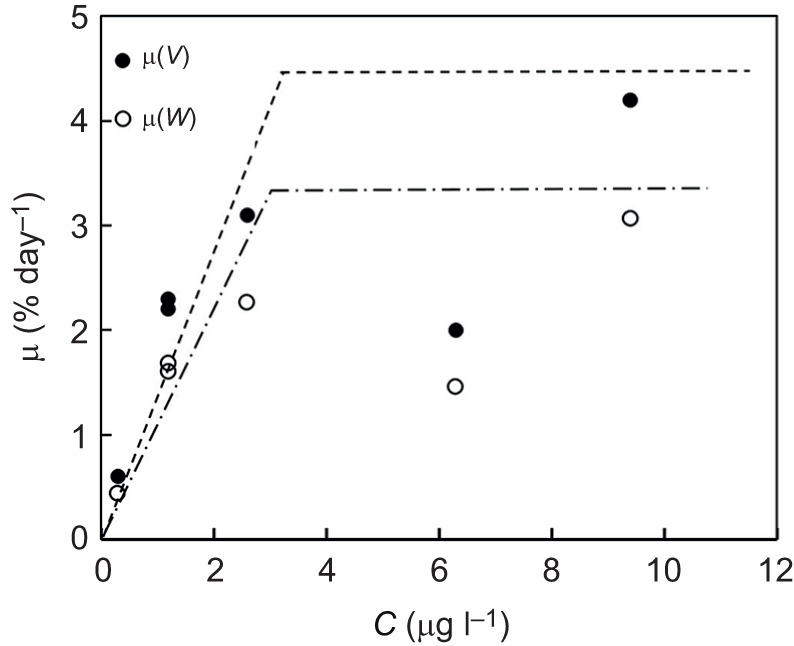
**Growth of *H. panicea* sponge module versus concentration of Chl *a*.** Volume-specific and weight-specific growth rates, µ(*V*) and µ(*W*), versus concentration of Chl *a* (*C*). Data from Table 2. Dashed and dash-dotted lines suggest possible trends consisting of linear increases to a maximal value.

Table 1 shows a summary of the key parameters of the growth experiments. Fig. 1 is an example of the growth of a sponge module fed with a certain algal concentration for 38 days and photographed weekly to determine the parameters needed for estimating module volume (*V*_mod_) according to Eqn 3. The growth history of the total sponge module volume (*V*) under different food treatments is shown in Fig. 2 and the corresponding regression equations are listed in Table 1. It can be seen that the growth rate increases with increasing algal concentration, with a maximum mean volume-specific growth rate of 4.2% day^−1^. The relationship between volume-specific filtration rate and total volume of sponge modules is shown in Fig. 3, where the power function relationship *F*/*V*=7.08*V*^–0.24^ is not far from the theoretical scaling relationship *F*/*V*∝*V*^−1/3^ described in the Introduction, although the power curve fit (*R*^2^=0.211) to the scattered data is less successful than a simple but theoretically unfounded linear regression line (*R*^2^=0.327). The relationship between the dry and wet weights of sponge modules is shown in Fig. 4. The power function *W*=0.047*W*_wet_^0.73^ indicates that an increasing part of the module volume is made up of water as the module grows bigger, which was used in deriving the scaling relationship for *F*(*V*). Further, using the power function for *W*, measured filtration and respiration data can be expressed in the usual way in terms of dry weight rather than volume. As shown in Fig. 5, *F*=*a*_1_*W*^b_1_^, *b*_1_=1.047 and *R*=*a*_2_*W*^b_2_^, *b*_2_=0.682, which suggests that *b*_1_≈1 (in agreement with the working hypothesis) and that *b*_2_<1. The data in Fig. 6 were obtained by applying Eqns 4 and 5 to dry weight data taken from converted total volumes in Table 2. The short time records of only three dates led to only two points at each food treatment in the plot of weight-specific growth rate µ=(1/*W*)d*W*/d*t*=*aW^b^* shown in Fig. S2, hence a considerable uncertainty, yet the expected trend of increasing µ with increasing Chl *a* concentration (*C*) is clear. A plot of µ versus *C* (Fig. 6) suggests a trend towards an upper maximum at high values of *C*.

**Table 2.**
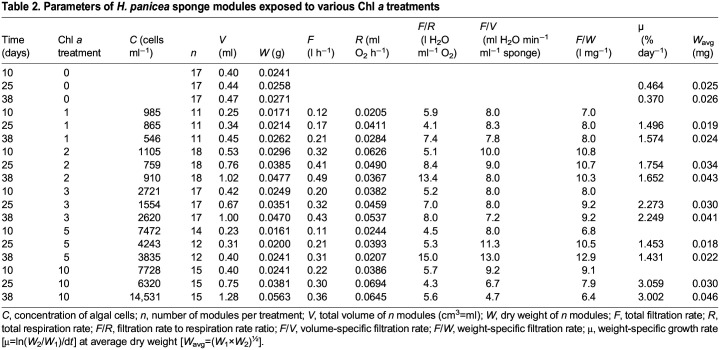
Parameters of *H. panicea* sponge modules exposed to various Chl *a* treatments

**Fig. 7.**
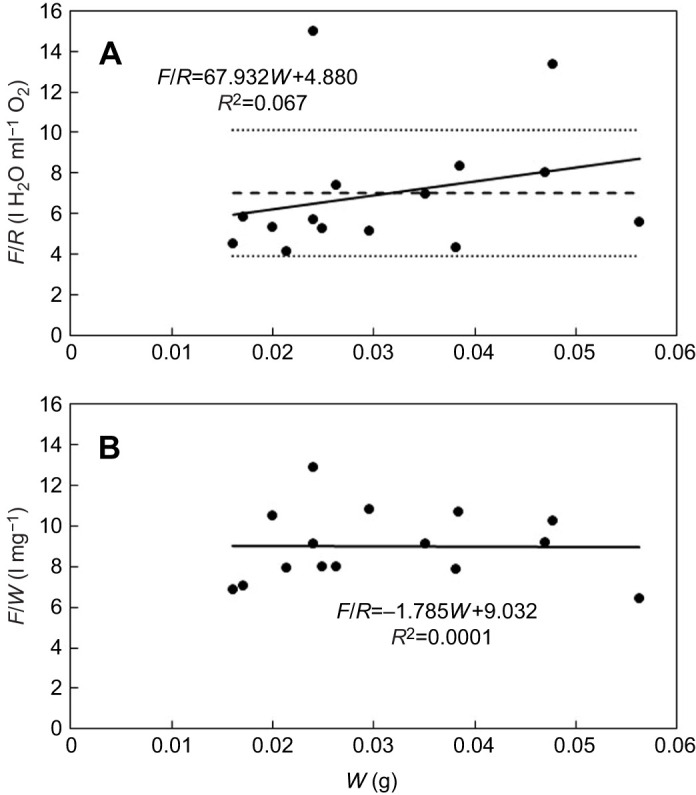
***F*/*R* ratio and *F*/*W* ratio versus dry weight of *H. panicea* sponge modules.** (A) *F*/*R* ratio as a function of the total dry weight of the module (*W*) and the associated regression line and equation. The dashed and dotted lines indicate the mean±s.d. (7.0±3.1 l H_2_O ml^−1^ O_2_). (B) *F*/*W* ratio as a function of *W*. Data from Table 2.

The *F*/*R* ratio versus dry weight (*W*) is shown in Fig. 7A and the *F*/*W* ratio versus *W* in Fig. 7B. The very low *R*^2^ value of 0.067 in Fig. 7A indicates no relationship, i.e. the *F*/*R* ratio is constant and independent of sponge size in agreement with the hypothesis for filter-feeding marine invertebrates that *b*_1_≈*b*_2_, and further, in the present case of a small growing sponge modules, that *b*_1_≈*b*_2_≈1*.* Thisis also confirmed by the lack of a relationship between *F*/*W* and *W* (Fig. 7B).

## DISCUSSION

In the present study, the mean *F*/*R* ratio was found to be 7.0±3.1 l of water filtered per millilitre of oxygen respired, which may be compared to a value of 11 reported by Riisgård et al. (1993), 2.7 reported by Thomassen and Riisgård (1995) and 15.5 l H_2_O ml^−1^ O_2_ reported by Riisgård et al. (2016). This last value indicates that the *F*/*R* ratio of mature *H. panicea* is generally above the minimum reference value of 10 l H_2_O ml^−1^ O_2_, but also that the ratio may be influenced by the experimental conditions, e.g. disturbance, water quality and food concentration. Thus, spontaneous contractions causing variations in filtration activity of single-osculum explants of *H. panicea* have been documented by, for example, Goldstein et al. (2019) and Kumala et al. (2023). In the present study, where the mean filtration rate (*F*) was measured on up to 18 modules, spontaneous contractions among modules probably reduced the mean filtration rate, whereas the mean total respiration rate (*R*) remained more stable, resulting in some scatter in the *F*/*R* data shown in Fig. 7A.

The scaling relationship *F*/*V*∝*V*^–1/3^ of a single-osculum module derived by Riisgård and Larsen (2022b) was based on the suggestion that *F* is determined by the product of the filtration rate of each choanocyte chamber and their number, which is proportional to the wall area between the inhalant and exhalant canals. It appears from Fig. S2 that the exponent *b* in the growth relationship µ=*aW^b^* is close to zero, which according to the bioenergetic growth model (see Eqn 3 of Riisgård et al., 2014) indicates that µ≈constant, and therefore *b*_1_≈*b*_2_≈1, which is exponential growth.

It can be concluded that the present data support the theoretical relationship *F*/*V*∝*V*^–1/3^ and that *b*_1_≈*b*_2_≈1 in small growing single-osculum explants.

## Supplementary Material

10.1242/jexbio.247132_sup1 Supplementary information

## References

[JEB247132C1] Clausen, I. and Riisgård, H. U. (1996). Growth, filtration and respiration in the mussel *Mytilus edulis*: no regulation of the filter-pump to nutritional needs. *Mar. Ecol. Prog. Ser.* 141, 37-45. 10.3354/meps141037

[JEB247132C2] Coughlan, J. (1969). The estimation of filtering rate from the clearance of suspensions. *Mar. Biol.* 2, 356-358. 10.1007/BF00355716

[JEB247132C3] Dahihande, A. S. and Thakur, N. L. (2019). Temperature– and size–associated differences in the skeletal structures and osculum crosssectional area influence the pumping rate of contractile sponge *Cinachyrella* cf. *cavernosa*. *Mar. Ecol.* 40, e12565. 10.1111/maec.12565

[JEB247132C4] Ereskovskii, A. V. (2002). Problems of coloniality, modularity, and individuality in sponges and special features of their morphogeneses during growth and asexual reproduction. *Russ. J. Mar. Biol.* 29, 46-56.

[JEB247132C5] Fiore, C. L., Baker, D. M. and Lesser, M. P. (2013). Nitrogen biogeochemistry in the Caribbean sponge *Xestospongia muta*: A source or sink of dissolved inorganic nitrogen? *PLOS ONE* 8, e72961. 10.1371/journal.pone.0072961 23991166 PMC3753232

[JEB247132C6] Fry, W. G. (1970). The sponge as a population: A biometric approach. *Symp. Zool. Soc. Lond.* 25, 135-162.

[JEB247132C7] Goldstein, J., Riisgård, H. U. and Larsen, P. S. (2019). Exhalant jet speed of single-osculum explants of the demosponge *Halichondria panicea* and basic properties of the sponge-pump. *J. Exp. Mar. Biol. Ecol.* 511, 82-90. 10.1016/j.jembe.2018.11.009

[JEB247132C8] Jørgensen, C. B., Møhlenberg, F. and Sten-Knudsen, O. (1986). Nature of relation between ventilation and oxygen consumption in filter feeders. *Mar. Ecol. Prog. Ser.* 29, 73-78. 10.3354/meps029073

[JEB247132C9] Kealy, R. A., Busk, T., Goldstein, J., Larsen, P. S. and Riisgård, H. U. (2019). Hydrodynamic characteristics of aquiferous modules in the demosponge *Halichondria panicea*. *Mar. Biol. Res.* 15, 531-540.

[JEB247132C10] Kumala, L., Riisgård, H. U. and Canfield, D. E. (2017). Osculum dynamics and filtration activity in small single-osculum explants of the demosponge *Halichondria panicea*. *Mar. Ecol. Prog. Ser.* 572, 117-128. 10.3354/meps12155

[JEB247132C11] Kumala, L., Thomsen, M. and Canfield, D. E. (2023). Respiration kinetics and allometric scaling in the demosponge *Halichondria panicea*. *BMC Ecol. Evol.* 23, 1. 10.1186/s12862-022-02102-w 37726687 PMC10507823

[JEB247132C12] Lee, W. L., Reiswig, H. M., Austin, W. C. and Lundsten, L. (2012). An extraordinary new carnivorous sponge, *Chondrocladia lyra*, in the new subgenus *Symmetrocladia* (Demospongiae, Cladorhizidae), from off of northern California. USA. *Invert. Biol* 13, 259-284. 10.1111/ivb.12001

[JEB247132C13] Lesser, M. P. (2023). Size effects on pumping rates in high microbial versus low microbial abundance marine sponges. *Oceans* 4, 394-408. 10.3390/oceans4040027

[JEB247132C14] Ludeman, D. A., Reidenbach, M. A. and Leys, S. P. (2017). The energetic cost of filtration by demosponges and their behavioural response to ambient currents. *J. Exp. Biol.* 220, 995-1007. 10.1242/jeb.173849 28011822

[JEB247132C15] McMurray, S. E., Pawlik, J. R. and Finelli, C. M. (2014). Trait-mediated ecosystem impacts: how morphology and size affect pumping rates of the Caribbean giant barrel sponge. *Aquat. Biol.* 23, 1-13.

[JEB247132C116] Reiswig, H. M. (1971). *In situ* pumping activities of tropical Demospongiae. *Mar. Bio.* 9, 38-50.

[JEB247132C16] Reiswig, H. M. (1974). Water transport, respiration and energetics of three tropical marine sponges. *J. Exp. Mar. Biol. Ecol.* 14, 231-249. 10.1016/0022-0981(74)90005-7

[JEB247132C17] Reiswig, H. M. (1975). The aquiferous systems of three marine Demospongiae. *J. Morph.* 145, 493-502. 10.1002/jmor.1051450407 30309053

[JEB247132C18] Riisgård, H. U. (2001). On measurement of filtration rate in bivalves - the stony road to reliable data, review and interpretation. *Mar. Ecol. Prog. Ser.* 211, 275-291. 10.3354/meps211275

[JEB247132C19] Riisgård, H. U. and Larsen, P. S. (2022a). Actual and model-predicted growth of sponges – with a bioenergetic comparison to other filter-feeders. *J. Mar. Sci. Eng.* 10, 607. 10.3390/jmse10050607

[JEB247132C20] Riisgård, H. U. and Larsen, P. S. (2022b). Filtration rates and scaling in demosponges. *J. Mar. Sci. Eng.* 10, 643. 10.3390/jmse10050643

[JEB247132C21] Riisgård, H. U., Thomassen, S., Jakobsen, H., Weeks, J. and Larsen, P. S. (1993). Suspension feeding in marine sponges *Halichondria panicea* and *Haliclona urceolus*: effects of temperature on filtration rate and energy cost of pumping. *Mar. Ecol. Prog. Ser.* 96, 177-188. 10.3354/meps096177

[JEB247132C22] Riisgård, H. U., Lundgreen, K. and Larsen, P. S. (2014). Potential for production of ‘mini-mussels’ in Great Belt (Denmark) evaluated on basis of actual growth of young mussels *Mytilus edulis*. *Aquacult. Internat* 22, 859-885. 10.1007/s10499-013-9713-y

[JEB247132C23] Riisgård, H. U., Kumala, L. and Charitonidou, K. (2016). Using the *F*/*R*-ratio for an evaluation of the ability of the demosponge *Halichondria panicea* to nourish solely on phytoplankton versus free-living bacteria in the sea. *Mar. Biol. Res.* 12, 907-916.

[JEB247132C24] Southwell, M. W., Weisz, J. B., Martens, C. S. and Lindquist, N. (2008). *In situ* fluxes of dissolved inorganic nitrogen from the sponge community on Conch Reef, Key Largo, Florida. *Limnol. Oceanogr*. 53, 986-996. 10.4319/lo.2008.53.3.0986

[JEB247132C25] Thomassen, S. and Riisgård, H. U. (1995). Growth and energetics of the sponge *Halichondria panicea*. *Mar. Ecol. Prog. Ser.* 128, 239-246. 10.3354/meps128239

[JEB247132C26] Vacelet, J. and Boury-Esnault, N. (1995). Carnivorous sponges. *Nature (London)* 373, 333-335. 10.1038/373333a0

