# Supplementary material for: Growth, filtration and respiration characteristics of small single-osculum demosponge Halichondria panicea explants
Source: J Exp Biol. 2024 Apr 19;227(8):jeb247132. doi: 10.1242/jeb.247132 (PMC11058627; doi:10.1242/jeb.247132)
Supplement: Supplementary information [file jexbio-227-247132-s1.pdf]

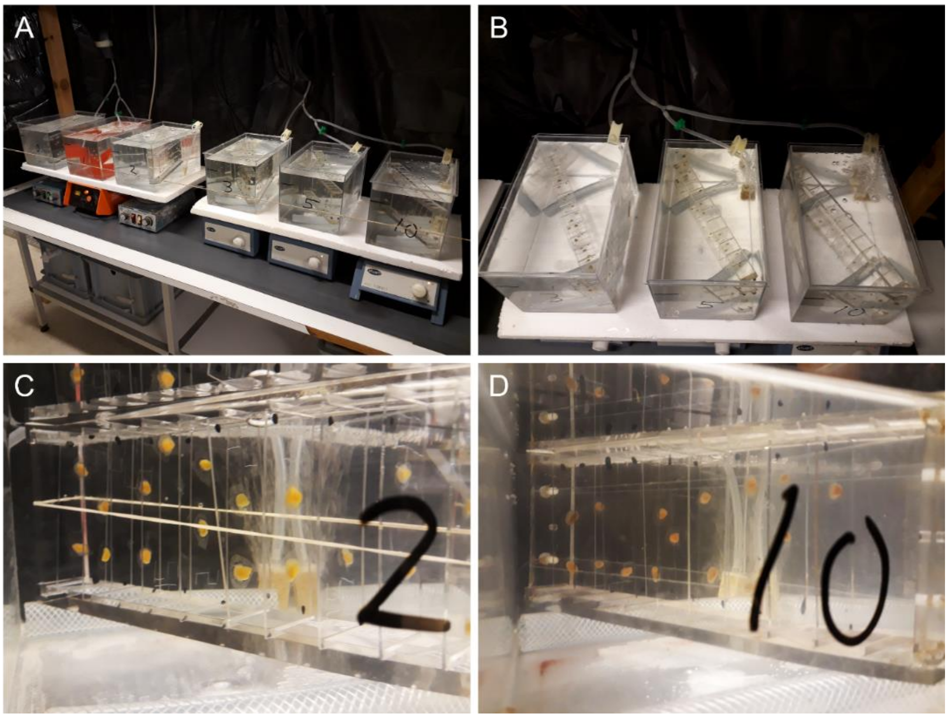

Fig. S1. A and B: Experimental feeding set-up of air-mixed seawater aquaria with *Halichondria panicea* explants (yellow spots on object slides) on magnetic stirrers. Numbers on aquaria indicate various food treatments (0, 1, 2, 3, 5, and 10 µg Chl *a* l^{-1}). C and D: Close-ups of treatment aquaria #2 and #10, respectively.

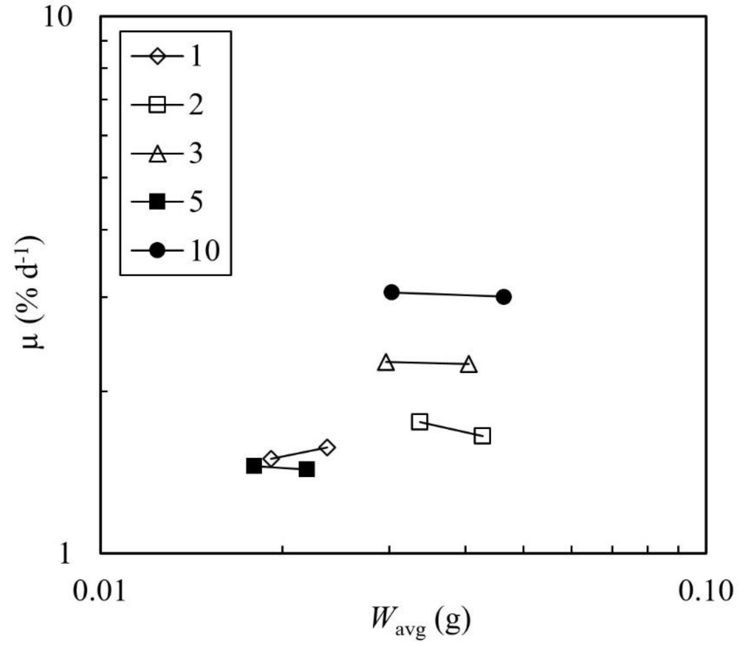

Fig. S2. *Halichondria panicea*. log growth rate (µ) versus log mean dry weight (W_{avg}). Data from Table 2.

Journal of Experimental Biology • Supplementary information

Table S1. ***Halichondria panicea*****. Growth experiments with various sponge modules in 6 food treatments**. r = module radius (mean ± SD), h = module height (mean ± SD) and V = total volume of all sponge modules per treatment (cf. Eq. 3) based on regular image analyses at time t = 0, 7, 15, 21, 28 and 38 d.

Chl *a* treatment	r_0 (mm)	r_7 (mm)	r_{15} (mm)	r_{21} (mm)	r_{28} (mm)	r_{38} (mm)
0	3.4±0.6	3.7±0.7	4.1±0.6	4.2±0.7	4.2±0.8	3.7±0.9
1	3.2±0.6	4.1±0.5	4.2±1.0	4.4±1.1	4.5±1.2	4.8±2.1
2	3.4±0.5	4.2±0.6	4.8±0.9	5.3±1.2	5.3±1.4	5.7±1.8
3	3.4±0.7	3.8±0.7	4.9±1.2	5.6±1.4	6.0±1.7	6.4±2.0
5	3.4±0.5	3.6±0.6	3.8±0.8	4.6±0.9	5.0±1.0	5.4±1.4
10	3.2±0.6	3.7±0.7	4.6±1.0	5.1±0.9	5.7±1.6	6.7±1.6
**Chl *a* treatment**	**h_0 (mm)**	**h_7 (mm)**	**h_{15} (mm)**	**h_{21} (mm)**	**h_{28} (mm)**	**h_{38} (mm)**
0	3.0±0.6	3.0±0.8	3.4±0.9	3.4±1.6	3.0±1.0	2.9±1.1
1	2.5±0.8	2.6±0.8	3.1±0.7	2.8±0.7	2.6±0.7	2.5±0.7
2	3.0±0.6	2.8±0.5	3.3±0.7	3.0±0.7	3.1±0.8	3.1±0.9
3	2.6±0.6	2.5±0.5	2.8±0.5	2.3±0.6	2.4±0.7	2.2±0.7
5	3.0±0.7	2.3±0.4	2.2±0.3	2.1±0.4	2.2±0.3	2.1±0.5
10	2.9±0.5	2.9±0.7	3.1±0.7	3.3±0.8	3.3±0.6	3.2±0.8
**Chl *a* treatment**	**V_0 (cm^3)**	**V_7 (cm^3)**	**V_{15} (cm^3)**	**V_{21} (cm^3)**	**V_{28} (cm^3)**	**V_{38} (cm^3)**
0	0.31	0.38	0.53	0.54	0.44	0.39
1	0.15	0.27	0.35	0.33	0.33	0.42
2	0.33	0.50	0.75	0.83	0.89	0.79
3	0.28	0.33	0.62	0.66	0.81	0.82
5	0.22	0.19	0.21	0.30	0.35	0.42
10	0.24	0.33	0.57	0.71	0.89	1.11

Journal of Experimental Biology • Supplementary information
